# Comparative phylotranscriptomics reveals ancestral and derived root nodule symbiosis programmes

**DOI:** 10.1038/s41477-023-01441-w

**Published:** 2023-06-15

**Authors:** Cyril Libourel, Jean Keller, Lukas Brichet, Anne-Claire Cazalé, Sébastien Carrère, Tatiana Vernié, Jean-Malo Couzigou, Caroline Callot, Isabelle Dufau, Stéphane Cauet, William Marande, Tabatha Bulach, Amandine Suin, Catherine Masson-Boivin, Philippe Remigi, Pierre-Marc Delaux, Delphine Capela

**Affiliations:** 1 Laboratoire de Recherche en Sciences Végétales (LRSV), Université de Toulouse, CNRS, UPS, Toulouse INP, Castanet-Tolosan, France; 2 LIPME, Université de Toulouse, INRAE, CNRS, Castanet-Tolosan, France; 3 INRAE, CNRGV French Plant Genomic Resource Center, Castanet-Tolosan, France; 4 INRAE, US1426, GeT-PlaGe, Genotoul, Castanet-Tolosan, France

**Keywords:** Plant evolution, Rhizobial symbiosis

## Abstract

Symbiotic interactions such as the nitrogen-fixing root nodule symbiosis (RNS) have structured ecosystems during the evolution of life. Here we aimed at reconstructing ancestral and intermediate steps that shaped RNS observed in extant flowering plants. We compared the symbiotic transcriptomic responses of nine host plants, including the mimosoid legume *Mimosa pudica* for which we assembled a chromosome-level genome. We reconstructed the ancestral RNS transcriptome composed of most known symbiotic genes together with hundreds of novel candidates. Cross-referencing with transcriptomic data in response to experimentally evolved bacterial strains with gradual symbiotic proficiencies, we found the response to bacterial signals, nodule infection, nodule organogenesis and nitrogen fixation to be ancestral. By contrast, the release of symbiosomes was associated with recently evolved genes encoding small proteins in each lineage. We demonstrate that the symbiotic response was mostly in place in the most recent common ancestor of the RNS-forming species more than 90 million years ago.

## Abstract (web summary)

The nitrogen-fixing root nodule symbiosis evolved more than 90 million years ago. This innovation emerged by rewiring existing genetic programmes. More recently, this symbiosis convergently gained evolutionary stability in two legume lineages.

## Main

Interactions between organisms form a continuum of associations, from parasitism to mutually beneficial symbioses^1^, which have contributed to the evolution and diversification of the plant lineage for billions of years^2^. The mutualistic symbioses formed with fungal or bacterial symbionts are associated with key ecological and evolutionary transitions, such as the colonization of land by plants 450 million years ago, which was enabled by the evolution of the arbuscular mycorrhizal symbiosis (AMS)^3,4^. Following this initial event, plants and their symbiotic partners have diversified, leading to the emergence of multiple types of mutualistic symbioses with microorganisms^2^. Two main groups of symbiotic associations can be distinguished: intracellular and extracellular symbioses. Extracellular symbioses include for instance plant–cyanobacteria interactions where the bacterial symbiont is hosted in dedicated canals and glands^5,6^, ectomycorrhizal symbioses between plant roots and ascomycete or basidiomycete fungi^7^, or the very specific association between *Dioscorea sansibarensis* and its bacterial symbiont restricted to leaf drip tips^8^. Intracellular symbioses in plants are mainly established with fungal symbionts^9^. The nitrogen-fixing root nodule symbiosis (RNS) is a rare example of intracellular accommodation of bacteria^9^. However, intracellular accommodation of symbionts during RNS occurs in different physical structures depending on the plant species, including transcellular tubular structures that retain bacteria^10^ or organelle-like structures called symbiosomes^11^ that completely release bacteria in host cells.

In extant species, RNS is found in ~17,500 species from four orders of flowering plants^12^: ~17,300 species from the Fabales and 230 species from the Fagales, Cucurbitales and Rosales, which together form the nitrogen-fixing nodulation (NFN) clade^13^. Comparative phylogenomic studies coupled with previous phylogenetic and physiological work provided insights into the evolutionary history of RNS. Although the original phylogenetic work^14^ and recent follow-up^15^ support convergent gains of RNS, the most likely scenario proposes that RNS was gained only once, before the diversification of the NFN clade. Following that single gain, RNS diversified in each lineage and was lost subsequently multiple times, leading to the scattered distribution observed in extant species^13,16,17^. The rate of RNS loss differs between lineages, with some displaying an evolutionarily stabilized association while others seem to have experienced massive losses^10^. However, the nature of the ancestral RNS, its functioning and how it diversified over 92–110 million years of evolution^12^ remain elusive.

The comparison of transcriptomic patterns across species in each context, whether developmental or in response to the environment, allows reconstruction of ancestral and derived responses to that context. For instance, this approach has been used in plants to reconstruct the flooding response in angiosperms^18^, to study the evolution of the shoot meristem^19^, organs and gametes^20^, and to infer the ancestral AMS transcriptome^4^.

Here we combine transcriptomics in multiple species and phylogenomics to reconstruct the ancestral RNS transcriptome. We further dissect the transcriptional shifts associated with each symbiotic step by exploiting experimentally evolved bacterial strains^21^, which progressively recapitulate the full symbiotic interaction. We use this combination of transcriptomics, phylogenomics and experimental evolution to reconstruct the evolution of the plant symbiotic programme.

## Results and discussion

### Identification of an ancestral RNS transcriptomic signature

The two largest groups of RNS-forming species, the Papilionoideae subfamily and the Mimosoid clade which is nested in the largely non-nodulating Caesalpinioideae subfamily^17,22^, belong to the Fabales order. While transcriptomic data have been obtained in response to RNS in a number of Papilionoideae^23–28^, the Mimosoids have been ignored. To fill this gap, we conducted a time-course experiment with *Mimosa pudica* inoculated with its bacterial symbiont *Cupriavidus taiwanensis* (Supplementary Table 1). As a preliminary to gene expression studies, we de novo sequenced the genome of *M. pudica*. This *Mimosa* species is tetraploid (2*n* = 4*x* = 52)^29^ and its genome size was previously estimated to be around 900 Mb (ref. ^16^). To generate a high-quality genomic sequence, we used a combination of long-read sequencing and optical mapping (see Methods) leading to a near-chromosome-level assembly (Supplementary Table 2). This method produced 74 hybrid scaffolds (from 128 kbp to 25.5 Mbp with N50 = 16.1 Mbp) for 52 expected chromosomes and a total genome size of 797.25 Mb. Automated structural annotation of the genome yielded 73,541 protein-coding genes and 5,134 non-coding RNAs. Finally, the high completeness of the annotated genome was evidenced by a 97% (2,255 genes) Busco recovery score on eudicots_odb10 (C:97.0% (S:10.2%, D:86.8%), F:1.1%, M:1.9%, *n*: 2,326). As expected for a tetraploid genome, most of the genes are duplicated. The expression of 51,214 *Mimosa* genes was detected in our complete transcriptomic dataset, 43% of which were differentially expressed (9,034 genes up/16,470 genes downregulated) during the symbiotic interaction with *C. taiwanensis* in at least one time point compared to non-inoculated roots (Supplementary Tables 3 and 4).

In addition, we generated the transcriptome of the Papilionoideae *Lupinus albus*, from the Genisteae tribe, inoculated with *Bradyrhizobium* sp. 1AE200 strain (Ledermann and Couzigou, unpublished). *L. albus* forms peculiar lupinoid nodules^30^ and its genome has been recently sequenced^31^. In brief, we identified 3,976/4,944 (up/down) differentially regulated genes in mature nodules in response to *Bradyrhizobium* sp. 1AE200 compared to non-inoculated roots (Supplementary Tables 1, 3 and 4). These differentially expressed genes (DEGs) represent around 33% of the 26,204 *L. albus* expressed genes.

To obtain comparable datasets, raw RNA-seq reads obtained in the presence or absence of their respective bacterial symbionts from seven other nodulating species (Supplementary Table 1) were remapped on their respective genomes, and differentially expressed genes were computed following the same approach as for *M. pudica* and *L. albus*. Due to sampling and sequencing depth heterogeneity among species, we used different fold-change thresholds to obtain comparable numbers of differentially expressed genes (see Methods). For each species, we also concatenated all differentially expressed genes at any time point to estimate the whole symbiotic response for up- and downregulated genes.

Between 2,275 (*Hippophae rhamnoides*) and 9,034 (*M. pudica*) differentially upregulated and 1,906 (*H. rhamnoides*) and 16,470 (*M. pudica*) downregulated genes were detected in the nine sampled species at any time of the symbiotic interaction (Supplementary Tables 3, 4 and 5). As expected, species for which transcriptomic responses were only analysed in mature nodules, such as *H. rhamnoides*, *Datisca glomerata* and *L. albus*, exhibited a lower proportion of differentially regulated genes (Supplementary Tables 3 and 5) as the earliest responses to the symbiont were probably not captured.

The observed massive symbiotic transcriptomic responses in each species reflect either a conserved response, species-specific responses or a mix of both patterns. To determine the evolutionary origin of these responses, we computed orthogroups^32^ for the nine studied species, together with 16 additional species from the NFN clade and *Arabidopsis thaliana* as outgroup (Supplementary Table 6). The additional species were chosen on the basis of genome quality and to cover RNS- and non-RNS-forming clades. The list of DEGs for each species was then cross-referenced with the list of genes present in each orthogroup. Using this approach, we were able to identify, for each species, the list of orthogroups containing at least one up- or downregulated gene.

**Fig. 1:**
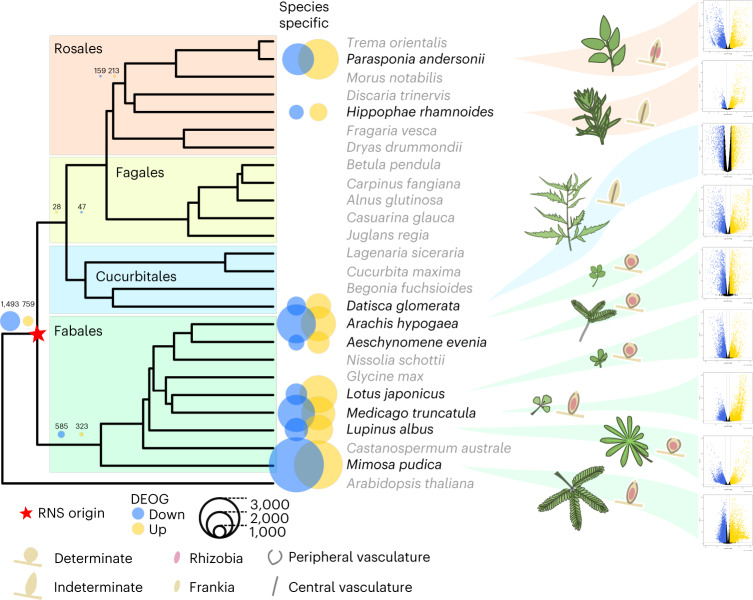
Conservation of the RNS transcriptomic response across NFN species. The tree depicts the orthofinder NFN phylogeny with *A. thaliana* as outgroup. Species used to compare symbiotic transcriptomes are indicated in black; species used to compute orthogroups are indicated in grey. The volcano plots on the right represent the logFC (*x* axis) by FDR *P* values (*y* axis) for the nine species at the latest time point. Blue and gold dots indicate significant downregulated and upregulated genes, respectively.

**Extended Data Fig. 1:**
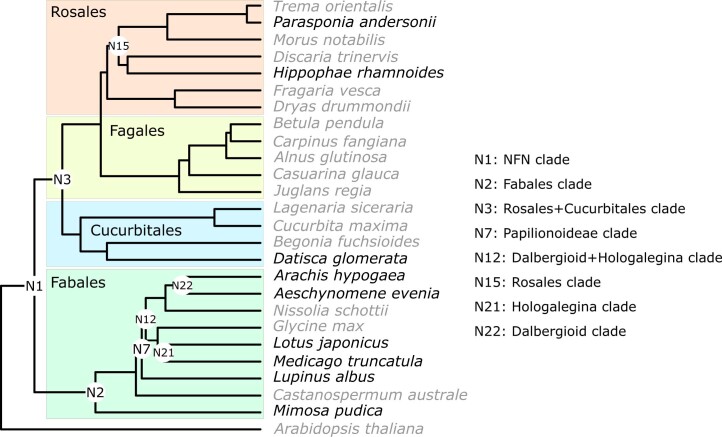
Simplified phylogeny to highlight node labels and species clades used in this study.

Using a simplified phylogeny composed of the nine species analysed here, we then mapped the presence/absence of each DEG-containing orthogroup (hereafter called DEOG for differentially expressed orthogroups) on each tip of the tree, allowing us to determine at which evolutionary node the genes have been recruited for RNS, using an ancestral state reconstruction of a discrete trait with a fixed-rates continuous-time Markov model. Using this method, we determined at which phylogenetic node the genes became differentially regulated during symbiosis (see Methods and Extended Data Fig. 1 for node label and position on the tree). Besides expression itself, the number of predicted DEOGs at a given node depends on several factors such as the maximum number of orthogroups present at that node or the accuracy of the orthogroup reconstruction method. To consider these biases, we assessed whether the experimentally determined values for each node significantly deviated from random expectation (see Methods). For each species, most of the genes were found to be differentially regulated in a species-specific manner (Fig. 1 and Supplementary Table 5). However, the observed numbers were either not significantly different from the null expectation, or were lower than expected (Supplementary Table 5). By contrast, a number of internal nodes displayed significantly more DEOGs than expected (Supplementary Table 5). In particular, 759/1,493 (up/down) orthogroups (211%/268% increase compared to the mean null expectation, Supplementary Table 5) were inferred to have been already up/downregulated in the most recent common ancestor of all RNS-forming species. Among the 759 ancestrally upregulated orthogroups, 157 contain genes with a known function, such as the Nod-factor receptor *LjNFR5*/*MtNFP*^33,34^, members of the downstream signalling pathway *SYMRK*/*DMI2* (refs. ^35,36^), *CCaMK*/*DMI3* (ref. ^37^) and *CYCLOPS*/*IPD3* (ref. ^38^), the master symbiotic regulator *NIN*^39^ or the infection-associated gene *RPG*^40,41^. The phylogenetic distribution of three of them (*LjNFR5/MtNFP*, *NIN* and *RPG*) has been recently linked with the ability to form RNS in the NFN clade. Indeed, all three genes have been lost independently in multiple lineages no longer able to form RNS^16,17^. In addition to the known genes, 593 orthogroups with undescribed functions were detected, including 110 that are absent from at least one non-RNS-forming species and present in all RNS-forming species, and thus represent top candidates for subsequent investigation by reverse genetics (Fig. 1 and Supplementary Table 7).

Specifically, the combination of transcriptomic responses and phylogenomics allowed us to identify a set of 759 orthogroups considered as ancestrally upregulated during RNS. We also identified orthogroups and genes recruited during the diversification of RNS in independent lineages, a pattern reminiscent of the potentiation–actualization–refinement model proposed for the evolution of novel traits^42,43^.

### Transcriptomic recruitment from pre-existing processes

**Fig. 2:**
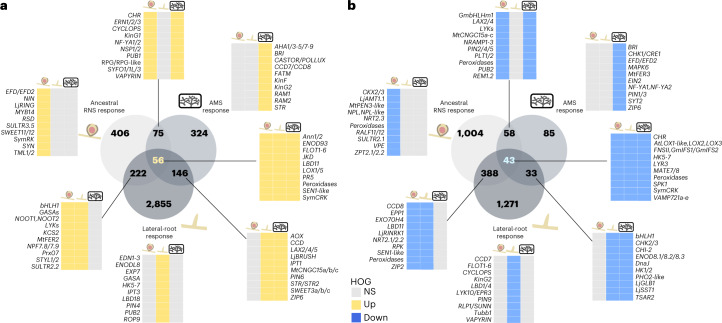
Non-proportional Venn diagrams presenting the shared and specific DEOGs among the ancestral RNS, AMS and lateral-root transcriptomic responses. Numbers in the Venn represent the number of DEOGs belonging to ancestral RNS, AMS and/or lateral-root response. **a**, Upregulated DEOGs. **b**, Downregulated DEOGs. NS, not significantly up/downregulated DEOGs. Heat maps represent a chosen list of DEOGs based on their role in RNS, AMS or lateral-root development.

Genetic dissection of RNS in model species proposed that its evolution relied on the co-option of genes regulating the more ancient AMS and lateral-root development^9,27^. To determine the contribution of these two programmes to the ancestral RNS transcriptomic response, we cross-referenced RNA-seq data obtained in the model legume *Medicago truncatula* for lateral-root development^27^ and AMS differentially regulated genes shared by *Lotus japonicus*^44^ and *M. truncatula*^45^ with the inferred ancestral transcriptome (Fig. 2 and Supplementary Table 7). Among the 759/1,493 orthogroups up/downregulated in the ancestral RNS transcriptome, 46%/33% behave like the lateral root, the AMS or both transcriptomic responses (Fig. 2 and Supplementary Table 7). Reversely, 54%/67% were specific to the RNS response, indicating that RNS evolved by combining the co-option of the older lateral-root and AMS programmes, but also via the recruitment of a substantial number of additional pathways.

### Transcriptomic rewiring by experimentally evolved symbionts

**Fig. 3:**
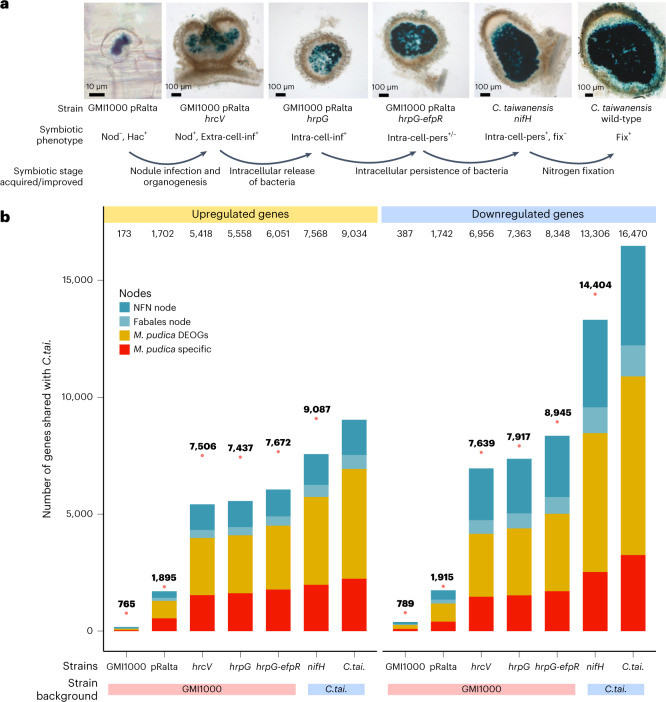
Genes recruited along the experimental evolution of RNS. **a**, Symbiotic phenotypes of *R. solanacearum* (GMI1000*)*-derived strains and *C. taiwanensis* symbionts. *M. pudica* plants were inoculated with *lacZ*-tagged strains and nodules were harvested at 10 dpi for *Ralstonia* and *C. taiwanensis nifH* mutants and at 14 dpi for *C. taiwanensis* wild-type (WT) strain. Roots and nodule sections were stained with X-gal. The *C. taiwanensis* WT picture is from ref. ^83^. Nod, nodule formation; Hac, root hair curling; Extra-cell-inf, extracellular infection of nodules; Intra-cell-inf, intracellular infection of nodules; Intra-cell-pers, intracellular persistence; Fix, nitrogen fixation. **b**, Number of genes up- and downregulated in nodules formed by the different *R. solanacearum* and *C. taiwanensis* mutants and shared with the symbiotic response obtained with the *C. taiwanensis* WT strain. The distribution of these genes in the NFN and Fabales nodes and in the *M. pudica* specific gene set is indicated. Pink dots and numbers above the bars indicate the total number of DEGs in each condition. *C.tai.*, *C. taiwanensis*.

RNS is a complex interaction involving multiple physiological and developmental processes that are often coupled and overlapping. In the case of most Fabales, these processes include the perception and response to the symbiotic signal produced by the symbionts (the so-called Nod-factors, NF), nodule organogenesis and the concomitant penetration of bacteria within root and nodule tissues, symbiosome release and persistence, and nitrogen fixation. The evolutionary transition from a non-RNS-forming state to a fully functional RNS state probably occurred over millions of years through a number of intermediate stages that cannot be captured in extant species. To define the transcriptional modules (and their evolutionary origin) associated with each process, we exploited a collection of bacterial mutants that gradually induce the full symbiotic programme. Most of these bacterial mutants originate from an evolution experiment that was developed to replay the evolution of symbiotic abilities in a legume symbiont^46–48^. In this evolution experiment, we first introduced the symbiotic plasmid pRalta from the rhizobium *C. taiwanensis* LMG19424 (refs. ^49–51^), one of many natural symbionts of *M. pudica*, into the non-symbiotic, soil-borne bacterium *Ralstonia solanacearum* GMI1000. Then, we propagated these chimaeric bacteria for 400 generations along successive nodulation cycles on *M. pudica*. Throughout the experiment, clones gradually gained symbiotic abilities^46,48,52^ and adaptive mutations responsible for the main phenotypic changes observed in the evolved clones were identified. RNS was obtained following mutations inactivating the Type Three Secretion System of *R. solanacearum*. A stop mutation in *hrcV*, a gene encoding a Type Three Secretion System structural protein, conferred to bacteria the capacity to nodulate *M. pudica* but nodules were only extracellularly invaded (Fig. 3a). By contrast, a stop mutation in *hrpG*, a gene encoding a global regulator of hundreds of genes including Type Three Secretion System genes, enabled bacteria to form nodules and invade them intracellularly through the formation of symbiosomes, which are released in the cytoplasm of nodule cells^46^. However, *hrpG* mutants degenerate very rapidly following symbiosome release (Fig. 3a). Cumulating an *hrpG* mutation with a mutation in the regulator *efpR* enhanced symbiosome persistence of bacteria although to a level not yet equivalent to a wild-type or a non-fixing mutant of *C. taiwanensis*^48^ and was not yet sufficient to enable nitrogen fixation in interaction with *M. pudica*. We reconstructed the adaptive mutations *hrcV*, *hrpG* and *hrpG-efpR* in the non-symbiotic original GMI1000+pRalta strain to generate a collection of nearly isogenic strains with increased symbiotic abilities (Fig. 3a). We analysed the transcriptome of *M. pudica* in response to inoculation with each of these three mutants, as well as with the non-nodulating parental strains GMI1000, GMI1000+pRalta and an *nifH* mutant of *C. taiwanensis*, which is only affected in its ability to fix nitrogen^53^. We harvested tissue samples, either roots, nodule primordia or nodules, at different time points between 1 and 21 d after inoculation to capture the most advanced symbiotic response (that is, most advanced stage/organ development) induced by each mutant (Supplementary Table 1).

**Extended Data Fig. 2:**
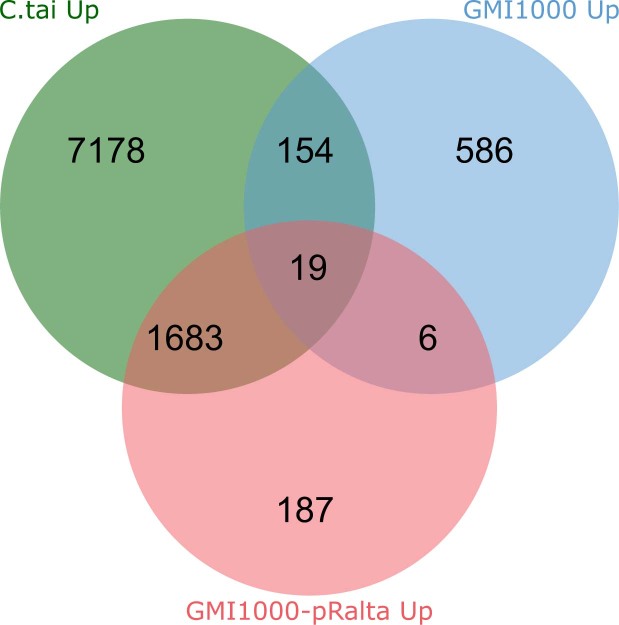
Venn diagram comparing the lists of up-regulated genes in response to *C. taiwanensis*, GMI1000 pRalta and GMI1000.

The evolution of improved symbiotic abilities in *Ralstonia* strains correlated with a gradual increase in the number of *M. pudica* DEGs that are also DEGs during the interaction with the wild-type *C. taiwanensis* strain (Fig. 3b). The gain of the symbiotic plasmid was sufficient on its own to activate 19%/10% (up/down) of the whole symbiotic response (Fig. 3b and Supplementary Table 4). Accompanying this gain of symbiotic response, the GMI1000+pRalta strain also did not activate the expression of 586 *M. pudica* genes specifically induced by the wild-type GMI1000 *R. solanacearum* strain (Supplementary Table 4 and Extended Data Fig. 2). A notable number of these genes are associated with the GO terms ‘oxido-reduction’, ‘cell wall organization’, ‘terpene synthase activity’, ‘diterpenoid biosynthetic process’, ‘gibberellin dioxygenase activity’ and ‘response to oxidative stress’, some of which may be involved in plant responses to microbial attack (Supplementary Table 8). This indicates that the horizontal gain of a symbiotic plasmid, a phenomenon widely observed within rhizobial populations^54^, may be sufficient to limit the activation of plant immunity. In the *Ralstonia hrcV* mutant forming extracellularly infected nodules, the transcriptomic response shared with the wild-type symbiont increased up to 60%/42% (up/down), while these proportions reached 66%/50% (up/down) with the *hrpG-efpR* strain. This pattern confirms phenotypic observations indicating that evolved *Ralstonia* strains are arrested at different stages along the progression towards a fully functional mutualistic state.

### The ancestral transcriptome supported multiple symbiotic traits

Next, we sought to trace the evolutionary history of transcriptomic recruitment or innovation associated with each of the following symbiotic traits: response to NF, nodule organogenesis and infection, symbiosome release, symbiosome persistence and nitrogen fixation. To do so, we exploited the gradual improvement in symbiotic abilities of *Ralstonia* strains and compared the responses of *M. pudica* to couples of strains that are able or unable to realize the different symbiotic traits (Fig. 3a and Supplementary Table 3). Transcriptomic responses to direct NF treatments were also available for two other Fabales, *M. truncatula* and *L. japonicus*^55,56^. Another dataset available for *M. truncatula* was obtained from laser-capture microdissection associated with nodule tissue differentiation, corresponding to symbiosome release (FIId) and symbiosome persistence (FIIp), plant and bacteroid cell differentiation (FIIp, IZ) and nitrogen fixation (ZIII) (Supplementary Tables 3 and 4)^57^. To consider genes related to the different traits, we focused on genes differentially regulated in the trait of interest in the same way as in the whole symbiotic transcriptomic response (Fig. 3, and Supplementary Tables 3 and 4). For example, genes upregulated for symbiosome release in *M. truncatula* have to be identified as upregulated in ‘FIId’ and in the whole symbiotic response RNS transcriptomic response of *M. truncatula*. As we have done above, all the genes linked with symbiotic traits were cross-referenced with orthogroups to infer when (that is, at which phylogenetic node) they were recruited for symbiosis during evolution.

**Fig. 4:**
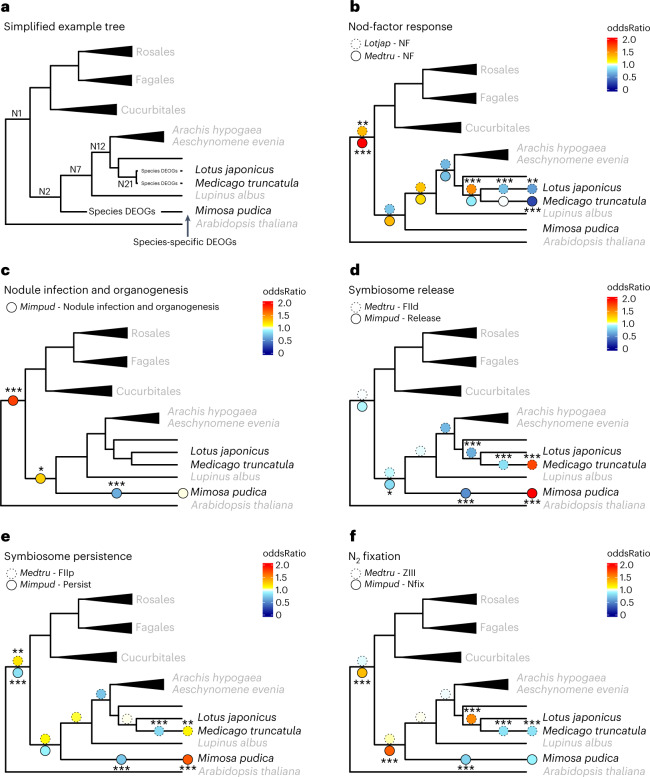
Evolutionary symbiotic-stage responses. Simplified phylogenies highlighting oddsRatios comparing the proportion of genes that are upregulated in the different symbiotic traits compared to the whole symbiotic response in the different evolutionary nodes. **a**, Simplified example tree. ‘Species-specific DEOGs’ represent HOGs in which only genes of a given species are present, while ‘Species DEOGs’ represent HOGs in which other species genes are present but differentially regulated in a species-specific manner or convergently acquired during symbiosis in this species. **b**, ‘Nod factor’ DEOGs oddsRatio for *L. japonicus* and *M. truncatula*. **c**, Nodule infection and organogenesis DEOGs oddsRatio for *M. pudica*. **d**, ‘Symbiosome release’ DEOGs oddsRatio for *M. truncatula* and *M. pudica*. **e**, ‘Symbiosome persistence’ DEOGs oddsRatio for *M. truncatula* and *M. pudica*. **f**, ‘N_2_ fixation’ DEOGs oddsRatio for *M. truncatula* and *M. pudica*. Asterisks indicate oddsRatios significantly different from ‘1’ using a two-sided Fisher’s exact test: *0.05 > *P* > 0.01, **0.01 > *P* > 0.001, ***P < 0.001; absence of symbols indicates non-significance. OddsRatios and corresponding *P* values are given in Supplementary Table 9.

The distributions of the gene sets for the different traits at the different evolutionary nodes were compared to the whole symbiotic transcriptomic response (Fig. 3). To do so, we used Fisher’s exact test to compare the whole symbiotic transcriptomic response and each trait node by node (Fig. 4 and Supplementary Table 9) to estimate over/under representation of genes in the different nodes. This analysis indicates that all stages of RNS involve genes that were already expressed in the most recent common ancestor of all RNS-forming species, although in different proportions, followed by different degrees of species-specific refinement.

An enrichment in ancestral genes was observed in the transcriptional responses associated with the perception of symbiont-produced Nod-factors in *M. truncatula* and *L. japonicus* as well as with nodule infection and organogenesis. In addition, both processes were linked with an impoverishment in species-specific DEOGs (Fig. 4b,c and Supplementary Table 9). Taken together, these results suggest that Nod-factor perception recruited ancestral DEOGs, followed by a large species-specific diversification facilitating recognition between symbiotic partners. Among the ancestral DEOGs in response to Nod-factors, we detected well-characterized NF-signalling components such as the transcription factors *NIN*^58,59^, *NF-YA1* (ref. ^60^), *NF-YA2* and *ERN1/2* (ref. ^61^), the infection genes *RPG*^40,41^, *VAPYRIN*^62,63^, *SYFO*^64^ and the LysM-RLK *EPR3/LYK10* (ref. ^65^), the LRR-RLK *RINRK1* (ref. ^66^) or the cytosolic kinase *SymCRK*^67^ (Fig. 4b, and Supplementary Tables 4 and 7). The chitinase *CHIT5* known to play a role in NF turnover in the Fabales *L. japonicus*^68^ was also found as part of this shared NF response, indicating that modulating NF levels was part of the ancestral RNS.

Organogenesis has been scrutinized in model legumes, revealing genes, in particular transcription factors, essential for the formation and maintenance of nodule identity^69^. Many of these transcription factors were recovered in the inferred ancestral transcriptomic signature of ‘organogenesis and infection’ (Fig. 4c, and Supplementary Tables 4 and 7). Expectedly, genes involved in this module partially overlap with the NF-responsive genes, including the master regulator *NIN* and its direct or indirect targets *RPG*, *NF-YA1*, *NF-YA2* and *ERN1/2*, while other NIN targets, such as the transcription factors of the *NF-YB* family, *LBD11* or *STY1/2* involved in the production of auxin maxima required for nodule primordium emergence^27,70^, specifically belong to the organogenesis and infection module (Fig. 4c). Another well-known transcription factor, *KNOX3*, regulating nodule development through activation of cytokinin biosynthesis but acting upstream of NIN, was found as part of this ancestral ‘organogenesis and infection’ programme^71^. Finally, *NOOT1* and *NOOT2*, which are known to maintain nodule identity in diverse legumes, were also detected^72^. Besides the known genes, 31 orthogroups annotated as transcription factors and so far not analysed in the context of RNS were detected. Their function during nodule organogenesis and infection remains to be determined.

Nitrogen fixation is a unifying feature of RNS. However, it has been predicted to be a trait that experienced important refinement during the diversification of the NFN. Indeed, mechanisms providing conditions for nitrogen fixation by the diverse symbionts in the nodules (*Frankia*, alpha- and beta-proteobacteria) vary notably^13^. Despite this diversification, our analysis revealed an over-representation of DEOGs associated with N_2_ fixation and linked with the ancestral RNS gene set for *M. pudica* and less species-specific DEOGs identified in both *M. pudica* ‘N_2_ fixation’ and the nodule ‘ZIII’ of *M. truncatula* (Fig. 4f and Supplementary Table 9). Most of these genes encode enzymes that have not been characterized yet (Supplementary Table 4).

Although symbiosome release, inferred from the *M. truncatula* (‘FIId’) and *M. pudica* (‘Release’) datasets, displays a peculiar evolutionary pattern (see below and Fig. 4d), this symbiotic stage also involved genes that are part of the ancestral transcriptomic response. Suppressors of plant defence in nodules, *SymCRK*^67^ and *RSD*^73^, as well as the transcription regulator *EFD* required for both plant and bacteroid differentiation in *M. truncatula*^74,75^ participate in this ancestral response. Looking specifically at the *M. pudica* data, we found *VAPYRIN*, *RPG*, some flotillin and remorin genes and the syntaxin *SYN*, which are well-known infection-associated genes^41,76–78^. We thus hypothesize that a proportion of genes linked with the ancestral RNS transcriptome and associated with ‘symbiosome release’ reflects infection (Fig. 4d).

As mentioned for the response to ‘Nod-factors’ and ‘infection and organogenesis’, DEOGs identified for the different traits often overlap, suggesting that genes such as transcription factors may act at different symbiotic stages^69^.

We identified the gene modules associated with ancient symbiotic processes including genes whose position in the symbiotic pathway remains to be characterized. Altogether, this indicates that the core mechanisms governing the response to ‘Nod factors’, nodule ‘infection and organogenesis’ and ‘nitrogen fixation’ in extant RNS-forming species have probably been conserved since their most recent common ancestor.

### Convergent evolution for symbiosome formation in legumes

By contrast with symbiont perception, nodule ‘infection and organogenesis’, and ‘nitrogen fixation’, the evolutionary pattern of ‘symbiosome release and persistence’ of rhizobia showed a decreased link with ancestral genes and an enrichment in species-specific DEOGs in both *M. pudica* (Caesalpinioideae) and *M. truncatula* (Papilionoideae, Fig. 4d,e and Supplementary Table 9). Compared to other orders of the NFN clade, RNS is evolutionarily stable in the Papilionoideae and the Mimosoid clade which is nested in the largely non-nodulating Caesalpinioideae subfamily^10^. It has been hypothesized that this stability is linked with the occurrence of symbiosome formation, which is almost exclusively found in these two clades^10^ and some species of the non-Mimosoid Caesalpinioideae genus *Chamaecrista*. Such a trait distribution might either reflect an ancestral gain in the Fabales and multiple subsequent losses or be the result of convergent evolution. The fact that the transcriptomic signature associated with that stage depends much more on genes regulated in a species-specific manner in both *M. truncatula* and *M. pudica* than the other ancestral traits strongly supports the hypothesis of convergent gains of symbiosome formation in the two lineages (Fig. 4d,e). This species-specific transcriptomic change may be the result of either the recruitment of existing genes into the symbiotic transcriptomic response or the de novo evolution of new genes in each lineage. To address this question, we analysed the nature of the upregulated genes associated with ‘symbiosome release’ and ‘FIId’ in a species-specific manner in *M. pudica* and *M. truncatula*.

**Fig. 5:**
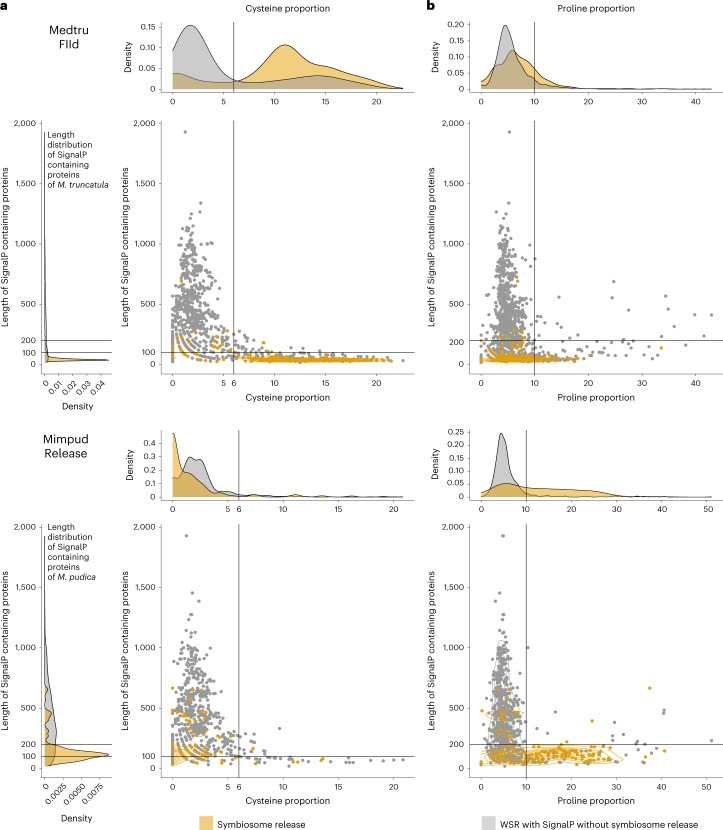
Characteristics of proteins with signal peptide from species-specific DEOGs related to symbiosome release in *M. truncatula* and *M. pudica*. **a**, Scatterplot representing proportion of cysteine (*x* axis) and protein length (*y* axis) for upregulated genes in the ‘whole symbiotic response’ (WSR) (grey) and ‘symbiosome release’ (gold). **b**, Scatterplot representing proportion of proline (*x* axis) and protein length (*y* axis) for upregulated genes in the ‘whole symbiotic response’ (grey) and ‘symbiosome release’ (gold).

First, we identified an over-representation of proteins with a predicted signal peptide in the ‘symbiosome release’ gene set (‘Release’ and ‘FIId’) compared with the ‘whole symbiotic response’ (without the genes tagged as related to ‘symbiosome release’) in both *M. pudica* (oddsRatio_Mimpud_signalP_ = 3.9; Supplementary Table 9) and *M. truncatula* (oddsRatio_Medtru_signalP_ = 4.2; Supplementary Table 9). Second, we wondered whether the proteins associated with ‘symbiosome release’ were different in size and/or amino acid composition from the proteins expressed during the whole symbiotic response. We observed that proteins associated with ‘symbiosome release’ were significantly shorter for both *M. pudica* (mean_Release_ = 156.9 vs mean_Mimpud_ = 401.3, *t*-test *P* = 2.78 × 10^−61^; Fig. 5 and Supplementary Table 9) and *M. truncatula* (mean_FIId_ = 55.4 vs mean_Medtru_ = 306.6, *t*-test *P* = 1.8 × 10^−141^; Fig. 5 and Supplementary Table 9). In addition, we found that these proteins showed more proline residues in *M. pudica* (ratio_mean_Pro_prop_ = 2, *t*-test *P* = 1.67 × 10^−19^) and more cysteine residues in *M. truncatula* (ratio_mean_Cys_prop_ = 1.9, *t*-test *P* = 1.0 × 10^−39^; Fig. 5a,b, and Supplementary Tables 9 and 10). Following these trends, we observed an enrichment in species-specific orthogroups (that is, only the sequence of the given species is present in the orthogroup) for cysteine-rich and small proteins in *M. truncatula*, but not for *M. pudica* (Fig. 5a and Supplementary Table 9). In *M. truncatula*, ‘symbiosome release’ is partly mediated by small proteins with a signal peptide and containing a high proportion of cysteine known as ‘nodule cysteine-rich’ (NCR) peptides (Fig. 5a). In inverted repeat-lacking clade (IRLC) and some dalbergioid legumes, these small secreted peptides have been shown to trigger the terminal differentiation of the nitrogen-fixing symbionts via antimicrobial activities preventing bacteroid proliferation outside the plant^79,80^. These NCRs correspond to the species-specific genes identified here for *M. truncatula*. Reversely, we observed an enrichment in species-specific orthogroups for proline-rich and small proteins in *M. pudica*, but not for *M. truncatula* (Fig. 5b and Supplementary Table 9). Proline-rich peptides have been found in insects, mammals and plants where they play a role as antimicrobial compounds^81,82^. Although *M. pudica* symbionts are not terminally differentiated, the revivability of *C. taiwanensis* bacteroids outside the plant is limited to 20%, indicating a possible intermediary state of differentiation^83^. The actual function of these proline-rich short proteins remains to be determined. Additionally, we looked at the evolutionary pattern of the *Arachis hypogaea* cysteine-rich secretory protein, antigen 5, and pathogenesis-related 1 proteins (*AhCAPs*) identified recently^24^. Of a total of 48 identified *AhCAPs*, 44 belong to *A. hypogaea* species-specific HOGs, suggesting another convergent evolution of secreted peptides linked to symbiosomes. Altogether, the presented data support the idea that convergence in the symbiosome release of symbionts evolved by at least two independent but analogous molecular processes: the de novo evolution of nodule-induced small proteins already proposed in dalbergioid^24^ and IRLC^80^.

## Conclusion

From the distribution of the trait and phylogenomic analyses, the leading hypothesis for the origin of RNS is that it was gained before the radiation of the NFN clade more than 90 million years ago^13,16^. Here we propose that RNS in the most recent common ancestor looked very similar to RNS in extant species. With the ancestral RNS transcriptomic signature now defined, future studies will have to decipher how this state evolved from a non-RNS-forming state. A role for the common symbiosis pathway in that process can be anticipated given its phylogenetic link with all the intracellular plant symbioses^84^ and the reverse genetic data obtained in diverse RNS-forming species^9^. The gain of a regulatory link between the common symbiosis pathway and the central RNS-regulator NIN at the base of the NFN clade represents one of the events that have played a role in the transition from the non-NFN-forming to the NFN-forming state^85^. For millions of years, RNS has been maintained in diverse lineages of the Fagales, Fabales, Cucurbitales and Rosales, with presumably very high rates of symbiosis loss. RNS has become evolutionarily stable in only two lineages: the Papillionideae subfamily and the Mimosoid clade in the Fabales order^10^. Our results support the hypothesis that evolutionary stability was acquired through the convergent evolution of symbiosome release and enhanced control of the bacterial symbiont gained by the expansion of putative antimicrobial peptide gene families. Besides providing an evolutionary perspective linked to multiple symbiotic traits and reconstructing a shared RNS transcriptomic response, our comparative transcriptomic approach has determined a list of conserved orthogroups that can be considered as targets for further spatial, temporal and biological characterization in multiple species. We believe that our dataset will help the community to integrate evolutionary perspectives in future studies. Candidate gene validation in multiple species would help in prioritizing key regulator genes for engineering nitrogen-fixing symbiosis in crops^86–88^.

## Methods

### *M. pudica* high molecular weight DNA extraction

High molecular weight (HMW) DNA was isolated from frozen young leaves using QIAGEN genomic-tips 100/G kit (10243) following the tissue extraction protocol. Briefly, 1 g of young leaf material was ground in liquid nitrogen with a mortar and pestle. After 3 h of lysis at 50 °C with proteinase K and one centrifugation step, the DNA was immobilized on the column. After several washing steps, DNA was eluted from the column, desalted and then concentrated by alcohol precipitation. The DNA was resuspended in TE buffer.

### *M. pudica* genome PacBio library preparation

A standard PacBio SMRTbell library was constructed from HMW DNA samples using the SMRTbell template prep kit 1.0 (Pacific Biosciences) according to PacBio recommendations (PN 100-938-400-03).

HMW DNA was sheared using a Megaruptor 2 system (Diagenode) to obtain a 40 Kb average size. Following an enzymatic treatment on 7.5 µg of sheared DNA sample for DNA damage repair, ligation with hairpin adapters to both ends of the targeted double-stranded DNA (dsDNA) molecule was performed to create a closed single-stranded circular DNA. A nuclease treatment was performed using a SMRTbell enzyme clean-up kit (Pacific Biosciences). Size selection with Blue-Pippin system (Sage Science) to remove fragments less than 15 Kb was done on purified sample with 0.45X AMPure PB beads (Pacific Biosciences). The size and concentration of the final library were assessed using the Fragment Analyzer system (Agilent) and the Qubit fluorometer and Qubit dsDNA HS reagents assay kit (Thermo Fisher), respectively.

Sequencing primer v3 and Sequel DNA Polymerase 3.0 were respectively annealed and bound to the SMRTbell library. The library was loaded on 8 SMRTcells 1M and sequencing was performed on the Sequel I system with Sequel sequencing kit 3.0, a run movie time of 600 min and Software v6.0 (PacBio).

### *M. pudica* genome assembly

The genome was assembled in three steps from PacBio reads. In Step 1, Canu v1.8 (ref. ^89^) was used to trim, correct and assemble the 5,815,198 subreads for a total size of 85 Gb, that is, an estimated coverage of 94X. Programme parameters were corOutCoverage=40, minReadLength=1000 and input genome size estimate=900 Mb. In Step 2, the raw data from PacBio Sequel bam files were aligned on the draft assembly. For this step, we used the wrapper of minimap2 (ref. ^90^), pbmm2, included in the SMRT Analysis Software v7.0.0 (https://www.pacb.com/products-and-services/analytical-software/smrt-analysis/). Programme parameters were « pbmm2 align–preset ‘SUBREAD’ -c 70 -l 500 ». In the final step (Step 3), we used this mapping result to polish the draft assembly and generate a high-quality final assembly. For this step, we used the variantCaller command included in the SMRT Analysis Software v7.0.0, with the arrow algorithm. Programme parameters were –algorithm arrow -minConfidence 40 -minCoverage 70 -coverage 100 -minReadScore 0.65. The final polished assembly produced had a total of 1,343 contigs with a total length of 842,189,795 bp, a largest contig of 24,170,175 bp and an N50 contig of 13,257,064 bp.

### Preparation of *M. pudica* ultra-high molecular weight

Bionano optical mapping was then used to further improve this assembly. Nuclei were purified from 0.5 g of dark treated young leaves according to the Bionano plant tissue DNA isolation base protocol (30068, Bionano Genomics), followed by ultra-high molecular weight (uHMW) DNA extraction based on the Bionano prep SP kit (80030, Bionano Genomics) adapted by our laboratory for plant samples. Briefly, plant leaves were flash frozen in liquid nitrogen and disrupted with a rotor-stator homogenizer (Qiagen). Nuclei were pelleted, washed and digested with proteinase K in lysis buffer. After phenylmethylsulfonyl fluoride treatment, a centrifugation step was added to eliminate cell wall debris. The supernatant was precipitated with isopropanol and captured with magnetic disk (Nanobind disk). After several washes, the uHMW DNA was eluted in elution buffer. Labelling and staining of the uHMW DNA were performed according to the Bionano prep direct label and stain protocol (30206, Bionano Genomics). Briefly, labelling was performed by incubating 750 ng of genomic DNA with DLE-1 enzyme (Bionano Genomics) for 2 h in the presence of DL-Green dye (Bionano Genomics). The DLE-1 enzyme recognizes the motif CTTAAG. Following proteinase K (Qiagen) digestion and non-fixed dye clean-up by membrane adsorption, the DNA backbone was stained with DNA Stain solution (Bionano Genomics) and incubated overnight at room temperature. The labelled DNA concentration was measured using Qubit dsDNA HS assay (Invitrogen).

### Data collection, optical mapping and genome scaffolding

Labelled DNA was loaded on a Saphyr G1 chip according to the Saphyr System user guide (30247, Bionano Genomics). Data processing was performed using the Bionano Genomics Access software (https://bionanogenomics.com/support-page/bionano-access-software/). Molecules (480 Gb) larger than 150 Kb with an N50 of 199 kbp were produced and represented 533X. This corresponded to 533X coverage of the 900 Mb estimated size of the *M. pudica* genome. These molecules were assembled using RefAligner with default parameters, producing 110 genome maps with an N50 of 16.1 Mbp for a total genome map length of 833 Mbp. Finally, hybrid scaffolding was performed between the polished PacBio assembly and the optical genome maps using hybridScaffold pipeline with default parameters. We obtained 74 hybrid scaffolds ranging from 128 kbp to 25.5 Mbp (total length 797 Mbp with N50 = 16.1 Mbp).

### *M. pudica* genome structural annotation

The *M. pudica* gene models were predicted by the eukaryotic genome annotation pipeline egn-ep (http://eugene.toulouse.inra.fr/Downloads/egnep-Linux-x86_64.1.5.1.tar.gz) using trained statistical models adapted for plants (http://eugene.toulouse.inra.fr/Downloads/WAM_plant.20180615.tar.gz). This pipeline manages automatically probabilistic sequence model training, genome masking, transcript and protein alignments computation, alternative splice sites detection and integrative gene modelling by the EuGene software (release 4.2a^91^; http://eugene.toulouse.inra.fr/Downloads/eugene-4.2a.tar.gz).

Four protein databases were used to detect translated regions: (1) the proteome of *M. truncatula* A17 (v5 annotation release 1.6; https://medicago.toulouse.inra.fr/MtrunA17r5.0-ANR/), (2) the proteome of the previous *M. pudica* Illumina genome^16^, (3) Swiss-Prot, October 2016 and (4) the proteome of *A. thaliana* TAIR10 version. Proteins similar to REPBASE were removed from the three datasets (to avoid the integration of transposable element related proteins in the training steps). Chained alignments spanning less than 50% of the length of the database protein were removed. The proteome of *M. truncatula* (release 1.6) was used as a training proteome by EuGene.

Three input transcripts for EuGene were used. One transcriptome was predicted on the basis of the mapping of reads from the 136 RNA-seq samples generated in this study (Supplementary Table 1).

To obtain these transcripts, the raw fastq paired-end reads were cleaned by removing the adapters and the low-quality sequences using cutadapt^92^ (v2.1) and TrimGalore (v0.6.5, https://github.com/FelixKrueger/TrimGalore) with the -q 30–length 20 options. The cleaned reads were mapped against the *M. pudica* genome assembly using HISAT2 (ref. ^93^) (v2.1.0) with the –score-min L,-0.6,-0.6–max-intronlen 10000–dta–rna-strandness RF options. Duplicated reads were removed using the SAMtools^94,95^ (v1.9) markdup command. Transcripts were predicted using Stringtie^96^ (v2.1.4) with –fr -f 0.8 on each sample. All 80 gtf sample files were merged using stringtie–merge with standard options. Transcript fasta files were generated using gffread^96,97^ (v0.11.6) with the -w option.

We also de novo predicted two transcriptomes from two batches of ten samples (one sample per condition) of our same RNA-seq data using DRAP pipeline^98^ (v1.92, http://www.sigenae.org/drap). runDrap was used on the 20 samples, applying the Oases RNA-seq assembly software^99^. runMeta was used to merge assemblies without redundancy on the basis of predicted transcripts with fpkm 1. These transcriptomes were employed as a training transcriptome by EuGene. Finally, 73,541 protein-coding genes, 1,107 transfer RNAs, 114 ribosomal RNAs and 3,913 ncRNAs were annotated.

Genome assembly, annotation file and gene models are publicly available through myGenomeBrowser^100^ and through NCBI under BioProject PRJNA787464.

### *M. pudica* RNA isolation and sequencing

*M. pudica* seedlings of Australian origin (B&T World Seeds, France) were grown in Gibson tubes containing nitrogen-free synthetic medium composed of a Fahraeus slant agar^101^ and liquid Jensen 1/4th medium^102^ at 28 °C and under a 16 h photoperiod as described previously^47^. *M. pudica* tissue samples were harvested at 1, 3 and 5 d post-inoculation (dpi) for non-inoculated plants, at 1 and 3 dpi for plants inoculated with non-nodulating *R. solanacearum* strains, at 1, 3, 5, 7 and 10 dpi for plants inoculated with *R. solanacearum* strains and at 1, 3, 5, 7, 14 and 21 dpi for plants inoculated with *C. taiwanensis* strains (Supplementary Table 1). Samples from four independent biological replicates were harvested at each time point. Samples from roots, nodule primordia and nodules were ground using a pestle and mortar before RNA extraction. Total RNA was isolated using the NucleoSpin RNA Plus kit (Macherey-Nagel) according to manufacturer’s instructions, treated with rDNase (Macherey-Nagel) for 10 min at 37 °C and then cleaned up with the NucleoSpin RNA clean-up kit (Macherey-Nagel). RNA quality was verified on a 2100 Bioanalyzer instrument (Agilent) and quantified on a QubitTM fluorometer (Thermo Fisher). RNA sequencing was performed at the GeT-PlaGe core facility, INRAE Toulouse. Polyadenylated messenger RNA and RNA-seq libraries were prepared according to Illumina’s protocols using the Illumina TruSeq Stranded mRNA sample prep kit to analyse mRNA. Briefly, mRNAs were selected using poly-T beads. Then, RNAs were fragmented to generate double-stranded complementary DNA and adaptors were ligated for sequencing. Eleven cycles of PCR were applied to amplify libraries. Library quality was assessed using a Fragment Analyser and libraries were quantified by qPCR using the Kapa library quantification kit. RNA-seq experiments were performed on an Illumina NovaSeq 6000 using a paired-end read length of 2 × 150 bp with the Illumina NovaSeq 6000 sequencing kits.

### *L. albus* RNA isolation and sequencing

For each biological replicate, *Bradyrhizobium* sp. 1AE200 (Ledermann and Couzigou, unpublished) strain was grown for 8 d on PSY medium agar plates (15 g l^−1^ bacto agar, DifcoTM, Becton Dickinson Bioscience) supplemented with erythromycin (200 µg ml^−1^) and 0.1% arabinose (w/v). Several independent colonies were used to inoculate a 20 ml PSY^103^ liquid culture supplemented with erythromycin (200 µg ml^−1^) and 0.1% arabinose (w/v) and grown in 100 ml Erlenmeyer flasks for 5 d at 28 °C under agitation (220 r.p.m.). Liquid culture was washed twice with 0.9% NaCl sterile solution (w/v) after centrifugation (10 min, 4,000 *g*). Bacterial suspension (1 ml, optical density (OD)_600_ = 0.05) was used to inoculate each seed.

*L. albus* cv. amiga seeds were sterilized using 4X diluted commercial bleach (9° Chl) for 2 min. Seeds were washed five times using sterile deionized water and spotted on 12-cm-diameter round petri dishes (nine seeds per dish) containing soft water agar medium (4.5 g l^−1^ bacto agar, Difco^TM^, Becton Dickinson Bioscience) for 3 d at 28 °C. Seeds with approximately 1-cm-long rootlets were planted in 200 ml glass jars containing sterile vermiculite and 100 ml of modified Jensen medium^104^ (in which no CaHPO_4_ was added and K_2_HPO_4_ was raised to 381 mg l^−1^). Seedlings were inoculated with *Bradyrhizobium* sp. 1AE200 or mock solution right after planting.

Plants were grown in a walk-in growth chamber illuminated with high-pressure sodium lamps (16 h photoperiod, 80% humidity, 26 °C and 22 °C day and night temperatures). For each biological replicate, at least eight independent root systems were used for collecting nodules or mock-inoculated root systems at 21 d after planting.

*L. albus* isolated nodules and root samples were harvested at 21 dpi for non-inoculated plants and inoculated plants with *Bradyrhizobium* sp. 1AE200 strain. Three biological replicates of inoculated and non-inoculated *Lupinus* root samples were used for RNA sequencing. Samples were ground using a pestle and mortar. RNA extraction and DNase treatment were performed respectively using E.Z.N.A. RNA extraction kit (Omega-Biotek) and TURBO DNA-free kit (Invitrogen) according to manufacturers’ instructions. Quality of RNAs was assessed using the Agilent 2100 Bioanalyzer system. RNA sequencing was performed by the Eurofins genomics facility. Polyadenylated mRNA and RNA-seq libraries were prepared according to Illumina’s protocols using the Illumina TruSeq Stranded mRNA sample prep kit to analyse mRNA. RNA-seq experiments were performed on an Illumina NovaSeq 6000 using a paired-end read length of 2 × 150 bp with the Illumina NovaSeq 6000 sequencing kits.

### Differential gene expression analysis

All RNA-seq libraries were mapped against their representative genome (Supplementary Table 1) using nextflow^105^ (v20.11.0-edge) and run on nf-core/rnaseq^106^ (v3.0, 10.5281/zenodo.1400710) using the ‘-profile debug,genotoul–skip_qc–aligner star_salmon’ options. The workflow used bedtools^107^ (v2.29.2), bioconductor-summarizedexperiment (v1.20.0), bioconductor-tximeta (v1.8.0), gffread^97^ (v0.12.1), picard (v2.23.9), salmon^108^ (v1.4.0), samtools^94^ (v1.10), star^109^ (v2.6.1d), stringtie^96^ (v2.1.4), Trimgalore (v0.6.6) and ucsc (v377). DEGs for the different species and experiments were estimated using ‘edgeR’^110^ in R^111^ (v4.1.2). Template script to estimate and identify DEGs is stored in GitHub at https://github.com/CyrilLibourel/Universal_nodulation_transcriptomic_response. Briefly, low-expressed genes with less than ten reads across each class of samples were removed. Then, gene counts were normalized by library size and using the trimmed mean of M-values normalization method^112^. We estimated DEGs by comparing symbiotic states to non-inoculated roots for the different species. *M. pudica* symbiotic traits (NF response, nodule organogenesis, symbiosome release and persistence and nitrogen fixation) DEGs were analysed with the DicoExpress tool^113^ that relies on the R packages ‘FactoMineR’^114^ and ‘edgeR’^110^ to identify genes that are differentially expressed between experimental conditions using generalized linear models. The lists of DEGs responsive genes as well as genes associated with the different symbiotic traits were determined by combining lists of DEGs originating from multiple comparisons between two samples as indicated in Supplementary Table 3. Genes were considered differentially expressed when the false discovery rate (FDR) was below 0.05 (Benjamini–Hochberg correction) and at a specific log fold-change (FC) (see Supplementary Table 3).

### Orthogroups reconstruction

To cross-reference expression data among species, we reconstructed orthogroups including the nine species for which RNA-seq data during symbiosis are available, as well as 16 representative species of each NFN clade and *A. thaliana* as outgroup (Supplementary Table 6). Orthogroups reconstruction was performed with OrthoFinder v2.5.2 (ref. ^115^) using the ultra-sensitive Diamond mode (-S diamond_ultra_sens option). The tree inferred by OrthoFinder was then checked for consistent reconstruction with known species trees and OrthoFinder reran on the basis of the species tree with the alignment and phylogeny methods using mafft v7.313 (ref. ^116^) and fasttree v2.1.10 (ref. ^117^), respectively, to infer hierarchical orthologous groups (HOG).

### Statistical analyses

The different scripts used to cross-reference orthogroups and DEGs to estimate differentially expressed orthogroups (DEOGs), reconstruct the ancestral transcriptomic symbiotic response, identify symbiosome accommodation proteins and all statistical related analyses are freely available at the dedicated GitHub repository ‘Universal_nodulation_transcriptomic_response’.

Briefly, to determine at which evolutionary node each orthogroup was probably transcriptionally recruited for RNS, we used the process described below:

1. DEGs to DEOGs: if at least one gene was up/downregulated in an orthogroup (HOG), the orthogroup was coded as 1 for the species in which the gene was differentially regulated. This process was done for the nine species in the 56,508 orthogroups.
2. On the basis of a simplified tree with only the nine species, we used the ‘asr_mk_model’ function from ‘castor’ package in R with the following options: ‘Nstates = 2, include_ancestral_likelihoods = TRUE, rate_model = ‘ER’’, giving a likelihood between 0 and 1 at each node.
3. To consider a DEOG to be recruited for RNS at a specific node, the likelihood had to be >0.6 to increase robustness for ancestral recruitment.
4. For each node ‘recruited’, we kept only the most ancient parental node. For example, if all nodes were ‘recruited’, the DEOG was considered as recruited in the common ancestor of NFN species (that is, NFN node). This method allowed us to accept ancestrally recruited HOGs that (1) were subsequently lost, (2) lost the deregulation during symbiosis or (3) for which information was missing because of heterogeneity of the sampling effort among species.
5. This process was conducted on all HOGs.

To estimate the null distribution of the number of DEOGs for each node, we first determined to which evolutionary node each orthogroup belongs using a maximum parsimony ancestral state reconstruction for discrete traits^118^ (that is, presence/absence of species genes in orthogroup). For each species, we randomly selected genes according to (1) the number of DEGs estimated in the species and (2) the node to which the orthogroup containing DEGs belongs. We repeated this process for the nine species and determined to which evolutionary node each orthogroup belongs using the same maximum parsimony ancestral state reconstruction (that is, presence/absence of species DEGs in orthogroup). We repeated this process 1,000 times to get the null expectation (Supplementary Table 5).

### Reporting summary

Further information on research design is available in the Nature Portfolio Reporting Summary linked to this article.

## Supplementary information

Reporting Summary

Supplementary Tables 1, 5, 6 and 8 Table 1. RNA-seq samples used in this study. Table 5. Observed and random expectation of DEOGs for each node. Table 6. Species used for orthogroup reconstruction. Table 8. GO terms enriched in the GMI1000-induced specific response.

Supplementary Table 2 Raw sequence data and statistics for *Mimosa pudica* genome and annotation completeness.

Supplementary Table 3 Sample comparisons to identify DEGs associated with the different symbiotic traits or during the symbiotic interaction in the different plant species. Tables with the number of up and downregulated genes by species associated with evolutionary node.

Supplementary Table 4 Tables for the nine species used in this study to summarize for each Gene_id, the HOG to which it belongs, the related evolutionary node in which HOG is attributed and the differential expression.

Supplementary Table 7 List of orthogroups and their evolutionary position where the DEOG is assigned.

Supplementary Table 9 Summary statistics of *t*-tests and Fisher tests.

Supplementary Table 10 *Medicago truncatula* and *Mimosa pudica* specific small secreted proteins associated with ‘symbiosome release’ (that is, ‘Mimpud Release’ and ‘Medtru FIId’).

## Data Availability

*Mimosa pudica* annotated genome assembly is available in myGenomeBrowser and raw sequenced data are available in NCBI under BioProject PRJNA787464 and SRP349803. All the repositories of RNA-seq used in this study are detailed in Supplementary Table 1. The orthogroups file generated is this study and raw gene count for the RNA-seq listed in Supplementary Table 2 are stored in figshare at https://figshare.com/projects/Comparative_phylotranscriptomics_reveals_ancestral_and_derived_root_nodule_symbiosis_programs/166196.

## Code availability

Scripts used to cross-reference orthogroups and RNA-seq data are publicly available in GitHub at https://github.com/CyrilLibourel/Universal_nodulation_transcriptomic_response
